# Supplementary figures and images for: The entropy rate of Linear Additive Markov Processes
Source: PLoS One. 2024 Apr 5;19(4):e0295074. doi: 10.1371/journal.pone.0295074 (PMC10997120; doi:10.1371/journal.pone.0295074)

(i) First-order Markov model

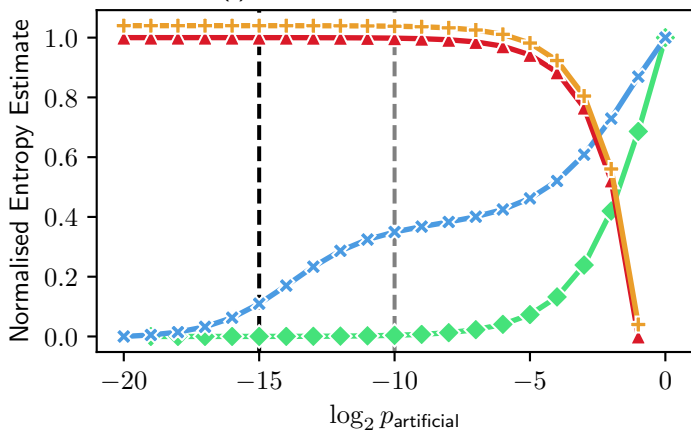

(ii) LAMP model

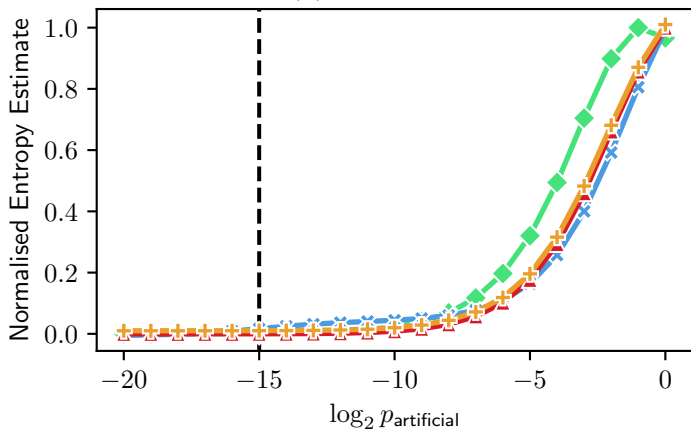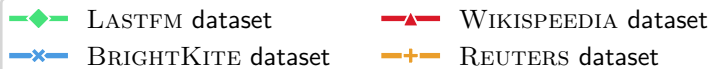

Supplement: S1 Fig — Plots to show convergence for the normalised entropy estimate value against log2 partificial, which was used to ensure ergodicity. (i) Shows hyperparameter sensitivity for the first-order Markov models and (ii) for the LAMP models. Each plot shows the effect of the weight of this artificial link on both the first-order Markov model estimate and the estimate obtained using the LAMP model for each dataset. We aim to find a region where the estimates are insensitive to the choice of hyperparameter. A dashed black line on each plot indicates the value when the artificial link weight is 2-15. This value was chosen as a global value, since it is a reasonable choice for all dataset model combinations, apart from the BrightKite dataset first-order Markov model, when a value of 2-10 was used to obtain the final estimate. This alternative value is indicated by a grey dashed line. The convergence curve for the WIKISPEEDIA and REUTERS datasets overlapped for the first-order Markov model, so the values for the REUTERS dataset was offset by +0.04 for visualisation. Small vertical offset was also added to the REUTERS and BRIGHTKITE datasets in the LAMP model visualisation (+0.01 and -0.005 respectively). (PDF) [file pone.0295074.s001.pdf]
