# Supplementary material for: The entropy rate of Linear Additive Markov Processes
Source: PLoS One. 2024 Apr 5;19(4):e0295074. doi: 10.1371/journal.pone.0295074 (PMC10997120; doi:10.1371/journal.pone.0295074)
Supplement: S1 File — (ZIP) [file pone.0295074.s002.zip › S1 Supporting information-1.pdf]

Supporting information

On connecting a Markov chain: Choosing artificial states and connection probabilities

For a unique stationary distribution of a Markov Chain to exist, it is necessary for the Markov Chain to be ergodic. By adding an artificial state to the chain, which is connected to all other states with some transition probability $p_{\text{artificial}}$, we can guarantee the chain is ergodic, and that a unique stationary distribution exists. The choice of hyperparameter $p_{\text{artificial}}$ is critical to ensuring this artificial state does not dramatically alter the behaviour of the Markov chain and instead acts as a link between otherwise disconnected communicating classes.

The parameter $p_{\text{artificial}}$ was chosen for each of the four datasets for both the LAMP and first-order Markov Chain approaches by calculating entropy estimates for $p_{\text{artificial}} = 2^{-i}$ for $i = 1, ..., 25$ for the first-order Markov estimates and $i = 1, ..., 50$ for the LAMP estimates. Since we are selecting for small values of $p_{\text{artificial}}$, it is also important to ensure we avoid numerical precision errors. It is important that the estimated value is robust to small changes in $p_{\text{artificial}}$. By selecting values of $p_{\text{artificial}}$ for which the estimate is stable and appears to have converged, we can estimate the entropy of the process. The true entropy value will differ between models and datasets, so we are not concerned with the value which the estimate converges to, only the stability of the estimate.

Generally, these estimates approach a stable value for larger values of i, although it is not known if this trend would continue, due to precision limitations. The largest value for which the estimate is stable was taken to perform each of the estimates was used.

The results of these simulations are shown in Figure S1. For all dataset model combinations except for the entropy estimates obtained using the first-order Markov model for the Wikispeedia and the Reuters datasets, the entropy values approach a limiting value from above, with most estimates converging to a stable value. The only estimate which does not appear to converge is the estimate obtained with the first-order model for the BrightKite dataset, which begins to flatten between -7 to -10, but then approaches zero. This may be due to numerical limitations, but this behaviour will be explored in future work.

In Figure S1, the entropy estimates are normalised to have a minimum value of 0 and a maximum value of 1, to enable the shape of the curves to be easily compared. This highlights the shape of the curve and emphasises the direction of convergence. These normalised convergence curves for the Wikispeedia and Reuters datasets overlap for the first-order Markov model, so the values for the Reuters dataset are offset by +0.04 for visualisation. Similarly, for the LAMP model convergence curves, a small offset is added to Reuters and BrightKite datasets in the LAMP model visualisation (+0.01 and -0.005 respectively).

The choice of $p_{\text{artificial}} = 2^{-15}$ appears appropriate for all datasets where the estimates appear to converge, while remaining large enough to avoid precision errors. This artificial transition probability is equivalent to observing a single transition amongst 32 768, which is sufficiently small to have a minimal impact on the scales we are considering. It is important that this choice is evaluated for different datasets, where the size of the data may make a different value more suitable.

Fig S1. Convergence for the entropy estimates on various datasets. Plots to show convergence for the normalised entropy estimate value against $\log_2 p_{\text{artificial}}$, which was used to ensure ergodicity. (i) Shows hyperparameter sensitivity for the first-order Markov models and (ii) for the LAMP models. Each plot shows the effect of the weight of this artificial link on both the first-order Markov model estimate and the estimate

obtained using the LAMP model for each dataset. We aim to find a region where the estimates are insensitive to the choice of hyperparameter. A dashed black line on each plot indicates the value when the artificial link weight is 2^{-15}. This value was chosen as a global value, since it is a reasonable choice for all dataset model combinations, apart from the BrightKite dataset first-order Markov model, when a value of 2^{-10} was used to obtain the final estimate. This alternative value is indicated by a grey dashed line. The convergence curve for the WIKISPEEDIA and REUTERS datasets overlapped for the first-order Markov model, so the values for the REUTERS dataset was offset by +0.04 for visualisation. Small vertical offset was also added to the REUTERS and BRIGHTKITE datasets in the LAMP model visualisation (+0.01 and -0.005 respectively).
